# Two Cases of Typhoid Fever

**Published:** 1857-03

**Authors:** Wm. H. Philpot

**Affiliations:** Redbone, Georgia


### ARTICLE II.

*Two Cases of Typhoid Fever.* By WM. H. PHILPOT, M. D., of Redbone, Ga.

Upon reading "The extracts from the Records of the Atlanta Medical Society," reported in your Journal (Dec. No.), I find a discussion upon the question, "Can we have Typhoid Fever with Intermittent Symptoms?"

It is, as remarked, a question of considerable importance; for, if we can determine in the outset, our case to be one of Typhoid Fever, it will certainly be of great assistance and utility in preventing and overcoming many difficulties in the treatment and favorable progress of the case. If, in every instance, the physician could, by a touch of the medulla oblongata, become satisfied he was to contend with this troublesome affection, it would be an event greatly to be desired, and of invaluable consequence to the practitioner. The pathognomonic symptom of my friend and preceptor, Prof. J. G. Westmoreland, I know, may exist—I have seen it exist, while by his side—I have examined cases when a student, and since then, but yet, I am certain I have seen cases of Typhoid Fever, and attended them, in which this tenderness in the region of the medulla oblongata was not present.

It is difficult to determine (at least I find it so), when calling to see a patient, the case to be one of Typhoid Fever, when we have presented as symptoms, strictly speaking, those of intermittent or remittent fevers. It is of importance to our patient, for us to distinguish, in the commencement, this first affection, from those of the two latter; for, in my experience, quinine is of no advantage in the treatment of this obscure disease, and much less so is mercury, for the increased nervous prostration, and irritability of the bowels, which they produce, will be the great objection to their use. The importance of distinguishing, in the outset, our case to be Typhoid Fever, has recently been indelibly impressed upon my mind, from treating two cases in which the symptoms were such as led to the treatment of remittent and intermittent fevers, until unmistakable symptoms made their appearance. I give you the cases:

I was called, Nov. 23d, 1856, to the plantation of Major

Daniel, to see a negro woman, Lucy, some, 30 years of age, who, the overseer informed me, had been sick some two or three days. Complained first, he said, of headache, and pains in her back and limbs; had had no operation for several days. He gave her, he informed me, "a dose of pills, warranted to cure fevers, liver complaint," &c., &c., but on the morrow found Lucy worse. The pills not operating, he repeated the dose, but she continued no better, and he sent for me.

I found Lucy with hot and dry skin; rapid and full pulse; tongue white, and furred; severe headache; some tenderness in epigastrium, and tenderness in lumbar spine; bowels costive; this was Sunday. On Friday, had high fever, she said, and very little on Saturday; every other day she was a great deal worse. Prescribed,

Hyd. Chlo. Nit.
Pul. Rheii. *aa* gr. x.
Pul. Ipecac, gr. iij.—Mix, to be taken at once; and

℞--Sulp. Quinine, gr. xx.
Pulvis Doveri, gr. xviij.

Make three powders—give one every four hours, commencing as soon as her fever abates.

24th.—Lucy is better—says it is her well day. Bowels moved once; commenced the quinine at 8 o'clock, A. M., yesterday, and took the last dose at sun-up this morning. No fever, she said, since some time between midnight and day, until then. 11 o'clock.—Her fever is returning; pulse full and strong, and 90 beats; nausea, and vomits a little; tenderness in the lumbar vertebræ, and in epigastrium. No tenderness in the region of the medulla oblongata, or in the right iliac region. Prescribed,

Sulp. Quinine, gr. xv.
Pul. Doveri, gr. xij.

Make three powders—one every three hours, commencing at 12, to-night, and to take, at once, a cathartic of hyd. chlo. mit. rhei. and ipecac.

25th.—Returned early this morning, to see my patient, and found her skin moist; pulse soft, and less frequent, being 70 beats to the minute; bowels moved once, occasioned by the medicine; no tenderness in epigastrium, iliac region, or of the medulla oblongata, but much less so in lumbar spine. Failing to cut short the disease, the paroxysms continuing to return, I determined to give the next dose of quinine myself. Prescribed,

Sulp. Quinine, gr. xx.
Pul. Doveri., gr. xviij.

Make three powders—one every three hours, commencing at 9 o'clock. After giving her one powder, left to attend to some other cases, and returned at 2 o'clock, on my way back to my office. Lucy's pulse was more full and strong; skin warm and dry; had taken the second powder at twelve; complained of some headache, and thought her fever was rising. I remained until 4 o'clock—prescribed, ¼ gr. of morphia to be taken at bedtime, and left Lucy with hot, dry skin; pulse 105 beats, and, on pressure, complained of some pain in the right iliac region; tongue not furred, with red edges, and no tenderness in the region of the medulla oblongata.

26th.—Lucy rested well last night after taking the morphia; complains of severe pain in right iliac region; pulse soft, feeble and frequent, 97 beats; skin slightly moist, and has great inclination to go to stool.

Prescribed morphia ½ gr., in table spoon brandy; left powder of morphia for bed time, and directed the brandy to be given every 5 or 6 hours.

27th.—Lucy is very feeble; pain in the iliac region is very great; pulse very feeble, and beats 107 to the minute; some tenderness in lumbar spine; none in medulla oblongata. Prescribed blister over iliac region, and continued the brandy; morphia power at night, and one in the meantime, if she is restless.

Thus was Lucy from day to day, with now and then some little diarrhœa, until December 15, when she commenced convalescing, and was discharged on the 17th.

The other case, Mrs. J———, of this county, who I was called to see on the 15th December, is an old lady, but has had excellent health; had no physician, she said, for quite a number of years before. She had been troubled for some days with some little cold and cough; slight headache and bowel complaint; had been to Major Daniels' plantation to see me for medicine, (his overseer's wife is her daughter,) but failed to see me, as I had left each time she came. She saw Lucy, and gave her several doses of medicine, while those who generally gave it were engaged. She continued to feel gradually worse, and at last took her bed and sent for me.

I found the old lady with hot and dry skin; pulse strong and full; thought she had a chill in the morning about 10 or 11 o'clock, and had had one every day for 3 days; tenderness in lumbar spine, and some pain in right side of abdomen; says the whole side pains her; no tenderness in the region of the medulla oblongata. Prescribed 3 powders of quinine and morphia, to be taken, one at day light, and one every 3 hours.

16th.—Found Mrs. J——— with high fever; great pain in the head; was sure she had a chill about 12 o'clock, although she did not shake. The pain in right side of abdomen is not so great; bowels not moved for two days; tenderness in lumbar and dorsal spine; none in medulla oblongata; always feels very well when her fever passes off. Prescribed 4 powders quinine and morphia, 6 gr. of the former and $\frac{1}{6}$ gr. latter; one to be taken at midnight, one at day light, and then one every 3 hours.

17th.—Mrs. J. thinks she had another chill about dinner time; at 4 o'clock her fever was high; skin dry and hot; tongue very dry and white furred. I discovered, on pressure, some pain in right iliac region, and says if *I bear hard*, her *neck* hurts her. Prescribed $\frac{1}{4}$ gr. morphia, and left a powder to be taken at night if she was restless.

18th.—Rested well last night, and feels better; if no chill to-day, says she will be up very soon; some tenderness in medulla oblongata, and in right iliac region; also tenderness of lumbar spine; has had two operations this morning mixed with blood; complains of being a little cold; gave her a tablespoon of brandy, and as it was near her chill time, waited to see if it would come on about one o'clock. She was certainly attacked with symptoms of a chill, which lasted some hour or more, when her pulse rapidly increased in number and some little in fullness, and her bowels were moved again. She having another bloody operation, prescribed—

Tinct. Catechu, ℥i.

Gum Mucilage, ℥i.

Sulphate Morphia, gr. iiij.

Mix—give a teaspoonfull, and repeat in a hour if another discharge; and after the next operation, brandy every 5 or 6 hours, and morphia powder during the night, if she should be restless.

19th.—Mrs. J. is not so well as yesterday; skin moist and pleasant; pulse some 80 beats; the pain in iliac region is quite severe, as also the pains in her head. She is restless, and talks in her sleep; is forgetful, and inclined to be delirous; had one operation during the night. Prescribed—

Aqua Camphor, ℥ss.

Morphia, gr. ss.

Mix—continue the brandy; and if another operation, give the catechu mixture.

Mrs. J. continued thus from day to day, sometimes with diarrhœa and delirium, until January 12th, when she became convalescent; and on the 16th was discharged.

